# Development of an interpretable machine learning model and web application for peri-colonoscopy hypoglycemia risk in hospitalized patients undergoing colonoscopy

**DOI:** 10.3389/fendo.2026.1903407

**Published:** 2026-07-20

**Authors:** Xiaodan Xu, Hang Zhao, Ganhong Wang, Kaijian Xia, Yu Ding, Jian Chen

**Affiliations:** 1 Department of Gastroenterology, Changshu No.1 People’s Hospital (Changshu Hospital Affiliated to Soochow University), Suzhou, China; 2 Department of Gastroenterology, Changshu Traditional Chinese Medicine Hospital (Changshu Hospital Affiliated to Nanjing University of Chinese Medicine), Suzhou, China; 3 Changshu Key Laboratory of Medical Artificial Intelligence and Big Data, Suzhou, China

**Keywords:** clinical prediction model, colonoscopy, machine learning, model interpretability, peri-procedural hypoglycemia

## Abstract

**Objective:**

To develop and externally validate a liquid neural network (LNN)-based model for predicting peri-procedural hypoglycemia in hospitalized patients undergoing colonoscopy, and to develop a cross-platform web application integrating real-time SHAP-based interpretability analysis.

**Methods:**

A total of 719 hospitalized patients undergoing colonoscopy were retrospectively enrolled from Changshu No.1 People’s Hospital and Changshu Traditional Chinese Medicine Hospital between January and December 2025, with peri-procedural hypoglycemia as the outcome and 28 candidate variables. Internal validation was performed using stratified five-fold cross-validation combined with out-of-fold (OOF) prediction, and LASSO feature selection and SMOTE class balancing were carried out within the training folds. Logistic regression (LR), decision tree (DCT), random forest (RF), extreme gradient boosting (XGBoost), and LNN models were constructed, and model performance was evaluated in terms of discrimination, calibration, and clinical utility; SHAP was used for global and individualized interpretation, and a web application was developed using Python–Streamlit.

**Results:**

The incidence of peri-procedural hypoglycemia was 15.2%. LASSO selected seven features: bowel preparation solution volume, sex, fasting duration, nutritional risk, insulin use, history of diabetes mellitus, and albumin. After SMOTE, the internal-validation AUCs in descending order were LNN 0.851 (95% CI: 0.804–0.893), RF 0.831, XGBoost 0.829, LR 0.765, and DCT 0.750; LNN simultaneously showed the best sensitivity–specificity balance (76.83%/85.50%) and the lowest Brier score (0.116), and decision curve analysis showed the highest net benefit across the 0.05–0.55 threshold range. The LNN-based web application achieved an AUC of 0.848 (95% CI: 0.758–0.921) in the external validation set of 168 patients, with a sensitivity of 74.07%, a specificity of 86.52%, and a negative predictive value of 94.57%; SHAP analysis identified nutritional risk (mean |SHAP| = 0.689), albumin (0.480), and sex (0.431) as the principal predictors.

**Conclusion:**

The LNN-based model can effectively assess the risk of peri-procedural hypoglycemia in hospitalized patients undergoing colonoscopy and maintained good predictive performance in external validation; the web application integrating real-time SHAP interpretation provides convenient, interpretable, individualized risk assessment, offering support for early screening and intervention decision-making. A publicly accessible demonstration of the web application is available at https://ml-model-for-hypoglycemia.streamlit.app/.

Colonoscopy is an important modality for colorectal cancer screening, diagnosis, and treatment, and its quality depends on adequate bowel preparation (1). However, the bowel preparation process can disturb peri-procedural glucose homeostasis and may precipitate adverse events such as hypoglycemia (2, 3). Hypoglycemia can cause palpitations, sweating, and impaired consciousness, and in severe cases may even compromise the safety and workflow of the examination. In hospitalized patients in particular, peri-procedural hypoglycemia may have tangible clinical consequences, including interruption or rescheduling of bowel preparation and the procedure, delayed post-procedural recovery, precipitation of cardiovascular events such as arrhythmia and myocardial ischemia, and prolonged hospitalization with increased medical costs (4, 5). It is therefore necessary to identify high-risk patients in advance during the bowel preparation phase.

Existing studies suggest that peri-colonoscopy hypoglycemia occurs at an appreciable rate and that the at-risk population is not limited to patients with diabetes. The incidence of peri-colonoscopy hypoglycemia in patients with type 2 diabetes mellitus may reach 18.7% (6), and the incidence during bowel preparation in patients with diabetes is approximately 16.8% (7); a survey of a general outpatient population likewise showed an incidence of approximately 17% during bowel preparation, associated with factors such as fasting time, subjective hunger, and anxiety level (8). These findings indicate that peri-procedural hypoglycemia is influenced by disease and medication, and is also closely related to bowel preparation behavior and nutritional reserve.

To date, predictive research on peri-colonoscopy hypoglycemia remains limited, and existing models have largely focused on patients with diabetes and relied predominantly on conventional logistic regression (6, 7). Beyond the reliance on a single algorithm, these models have several limitations for hospitalized patients undergoing colonoscopy: their study populations are typically restricted to patients with diabetes and do not cover non-diabetic inpatients who are nonetheless at risk (e.g., those with nutritional risk or prolonged fasting); they are usually derived under a single bowel-preparation regimen and seldom account for differences in the type and volume of cleansing agents; and they rarely address the calibration, clinical net benefit, interpretability, and external generalizability needed to inform peri-procedural clinical management. In the broader setting of hypoglycemia prediction, machine learning algorithms such as XGBoost, random forest, and CatBoost have demonstrated good discriminative performance (9–11); however, multi-algorithm comparison, probability calibration, clinical net-benefit assessment, interpretability analysis, and independent external validation in hospitalized patients undergoing colonoscopy remain insufficient.

The liquid neural network (LNN) is a class of continuous-time neural network models that can characterize complex nonlinear relationships through learnable time constants (12, 13) and has shown potential application value in clinical risk prediction research. Unlike conventional deep architectures such as multilayer perceptrons, which tend to overfit on small, low-event-rate tabular datasets, the input-dependent time constants of the LNN allow its hidden state to evolve dynamically and thereby capture high-order nonlinear interactions among variables (e.g., the joint effect of fasting duration, insulin use, and nutritional risk) with relatively few parameters. We therefore hypothesized that, even on static tabular data, the LNN could match or improve upon established algorithms such as XGBoost and random forest, while its continuous-time formulation also reserves a natural extension to future time-series inputs. Moreover, because the opaque, “black-box” nature of machine learning models can limit clinical trust and adoption, interpretability has become a prerequisite for clinical prediction models. SHapley Additive exPlanations (SHAP) can quantify the contribution of different variables to model output (14), helping to improve the transparency of machine learning models. On this basis, the present study first aimed to construct and compare multiple machine learning models and to systematically evaluate their performance using out-of-fold prediction, independent external validation, calibration curves, and decision curve analysis. Second, the study aimed to develop a cross-platform web application integrating real-time SHAP interpretation, so as to support early screening and individualized intervention for peri-procedural hypoglycemia in hospitalized patients undergoing colonoscopy.

## 1 Materials and methods

### 1.1 Study population and data collection

**Figure 1:**
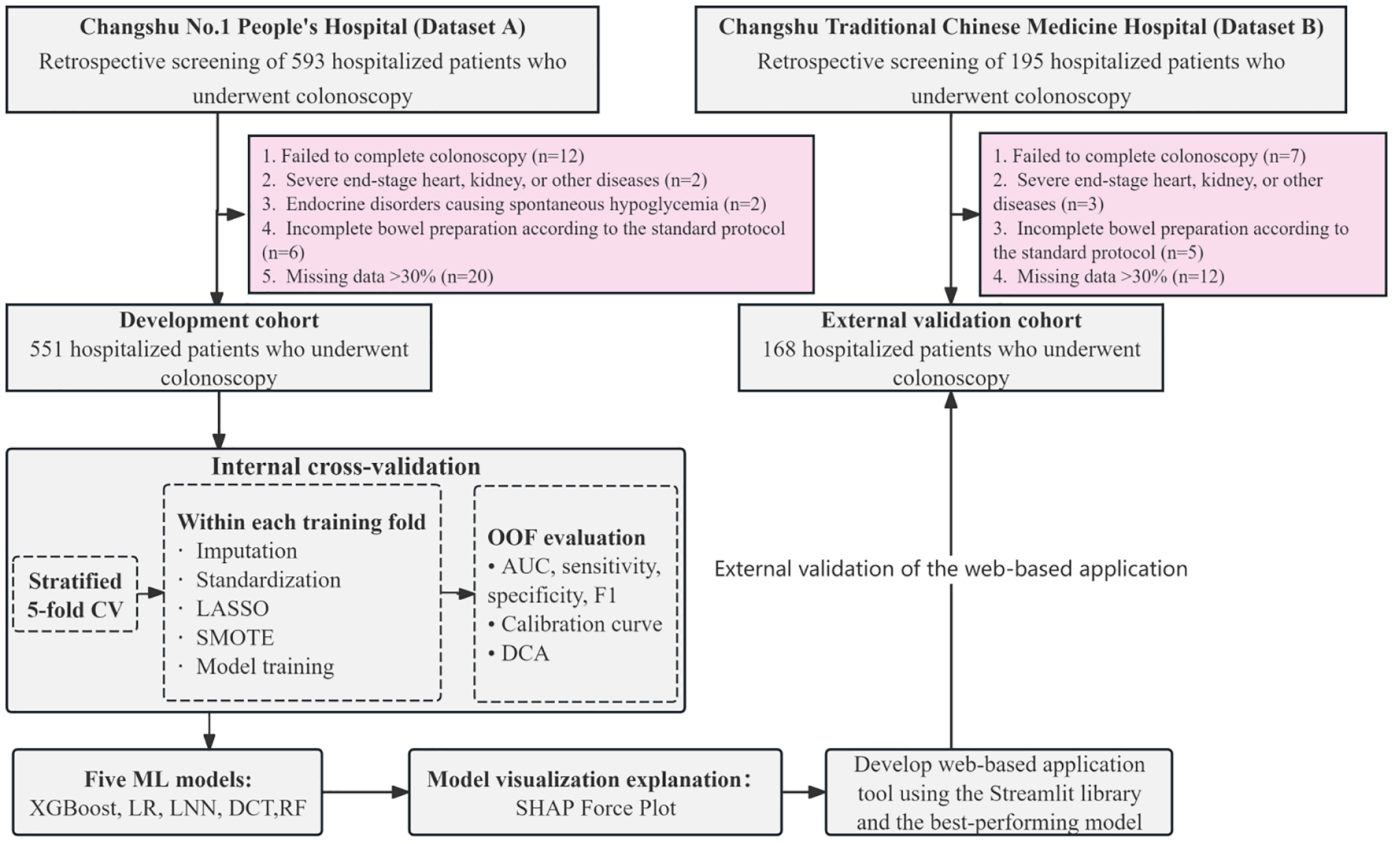
Research flowchart.

A total of 719 patients who were hospitalized and underwent colonoscopy at Changshu No.1 People’s Hospital (dataset A) and Changshu Traditional Chinese Medicine Hospital (dataset B) between January 2025 and December 2025 were retrospectively collected, comprising 109 patients in the hypoglycemia group and 610 in the non-hypoglycemia group. The two datasets were collected over the same calendar period but comprised independent, non-overlapping patient populations from two different hospitals, with no patient contributing to both datasets. Both centers applied the same standardized protocols, following the same ESGE-recommended bowel-preparation regimen and an identical peri-procedural blood glucose monitoring schedule. Dataset A served as the development set, used for model training, internal validation, and web application development; dataset B, from Changshu Traditional Chinese Medicine Hospital, served as an independent external validation set to evaluate the generalization performance of the model. The study design and workflow are shown in **Figure 1**.

Inclusion criteria: (1) age ≥ 18 years; (2) bowel preparation performed according to a standard bowel preparation regimen recommended by the European Society of Gastrointestinal Endoscopy (ESGE) guideline (1) (including the split-dose polyethylene glycol electrolyte solution regimen and other guideline-endorsed cleansing preparations). Exclusion criteria: (1) failure to complete colonoscopy owing to intestinal stenosis or other reasons; (2) comorbid end-stage disease such as severe cardiac, cerebral, pulmonary, hepatic, or renal failure; (3) known endocrine disorders causing spontaneous hypoglycemia, such as insulinoma; (4) emergency cases or patients who did not complete bowel preparation according to the standard regimen; (5) patients with > 30% missing clinical data.

### 1.2 Data collection and definitions

The features collected in this study included: (1) demographic characteristics: age, sex, and body mass index (BMI); (2) past and lifestyle history: history of diabetes mellitus (DM), coronary heart disease (CHD), previous colonoscopy, smoking history, and drinking history; (3) laboratory indices: albumin (ALB), fasting C-peptide, total bilirubin (TBIL), direct bilirubin (DBIL), aspartate aminotransferase (AST), alkaline phosphatase (ALP), total cholesterol, triglycerides (TG), blood urea nitrogen (BUN), creatinine (Cr), serum sodium (Na), uric acid (UA), and platelet count (PLT); (4) vital signs and anthropometric measurements: resting systolic blood pressure (resting SBP); and (5) colonoscopy- and peri-procedure-related factors: bowel preparation solution volume (BP volume), type of bowel preparation agent, fasting duration, nutritional risk, insulin use, and pre-procedure diet type.

Hypoglycemia determination and monitoring: In accordance with the internationally accepted standard (15), a blood glucose level ≤ 3.9 mmol/L (70 mg/dL) was defined as a hypoglycemic event, applied uniformly to patients with and without diabetes. This threshold served as the operational definition of a peri-procedural hypoglycemic event throughout the study and was applied consistently across all monitoring time points. Fingertip blood glucose was measured at the start of bowel preparation, at its completion, and 30 min before the resumption of eating; hypoglycemia at any time point was deemed a peri-colonoscopy hypoglycemic event. The monitoring window thus spanned the bowel-preparation phase through the immediate post-procedural period before refeeding; intra-procedural glucose was not separately monitored, as colonoscopy was completed within a short interval, frequently under sedation. Glucometers were quality-controlled and calibrated by dedicated personnel, and fingertip blood sampling and monitoring were performed by two trained and qualified nurses. Patients presenting with hypoglycemic symptoms such as palpitations, hand tremor, sweating, or pallor were immediately given glucose or sugar-containing food. NRS 2002 nutritional risk screening (16), the preferred screening tool recommended by ESPEN for hospitalized patients, comprises scores for disease severity, impaired nutritional status, and age (1 point added for age ≥ 70 years); the total score ranges from 0 to 7, with a score < 3 indicating no nutritional risk and a score ≥ 3 indicating nutritional risk.

### 1.3 Feature selection and model construction

Based on the development set, stratified five-fold cross-validation combined with out-of-fold (OOF) prediction was used for model development and internal validation. Patients with more than 30% missing clinical data were excluded. For the remaining sporadic missing values, continuous variables were imputed using the median, whereas categorical variables were imputed using the mode. All imputation parameters were estimated only within the training folds and were subsequently applied to the corresponding validation folds and the external validation set. The proportion of missing values for each predictor is reported in **Supplementary Table 1**. The development set was stratified by outcome event and randomly divided into five mutually exclusive subsets; four folds served as the training folds and the remaining fold as the validation fold, iterating cyclically over five rounds. To strictly prevent data leakage, missing-value imputation, min–max normalization, LASSO variable selection (determination of the λ value), and model training were all performed within the training folds only, and the resulting preprocessing parameters, feature subsets, and models were subsequently applied to the corresponding validation fold to output predicted probabilities. After the five iterations, the predicted probabilities from each fold were concatenated to form OOF results covering all development-set samples, which were used for internal-validation performance assessment.

Based on the features selected within each training fold, five predictive models—logistic regression (LR), decision tree (DCT), random forest (RF), extreme gradient boosting (XGBoost), and liquid neural network (LNN)—were constructed. To address class imbalance, SMOTE oversampling was performed independently within the training folds only and strictly after the train-validation split, while the validation folds retained their original distribution; Consequently, no synthetic samples could leak into the validation folds. The normalization parameters required by LR and LNN were likewise fitted only on the training folds, whereas DCT, RF, and XGBoost were not normalized. After internal validation, the preprocessing pipeline and LASSO selection were re-executed on the full development set in a single refit—rather than aggregating or pooling feature subsets across folds— to obtain the final fixed feature subset and to train the final model. Once the feature subset, preprocessing parameters, model parameters, and classification threshold of the final model were fixed, the entire pipeline was frozen. The frozen pipeline was then applied directly to the external validation set, which did not participate in any modeling step.

### 1.4 Model interpretation

To improve the transparency of the predictions made by the LNN model, the SHAP method was used to interpret the model output, focusing on the direction and relative magnitude of each variable’s contribution to the predicted probability of hypoglycemia (14). Because the LNN is not a tree-based model, the model-agnostic Kernel SHAP algorithm was used to compute SHAP values, with the background distribution defined as the entire development set. At the global level, SHAP values for each variable were calculated based on the predictions of the final LNN model on the external validation set, and a variable importance plot and a summary plot were generated, ranked by mean |SHAP value|, to identify the main driving factors and their directional effects. At the individual level, the seven input variables of a single patient were entered into the fixed LNN model, and the corresponding SHAP force plot was calculated and displayed to present the specific contribution of each variable in increasing or decreasing the predicted probability (17).

### 1.5 Web application development

**Figure 2:**
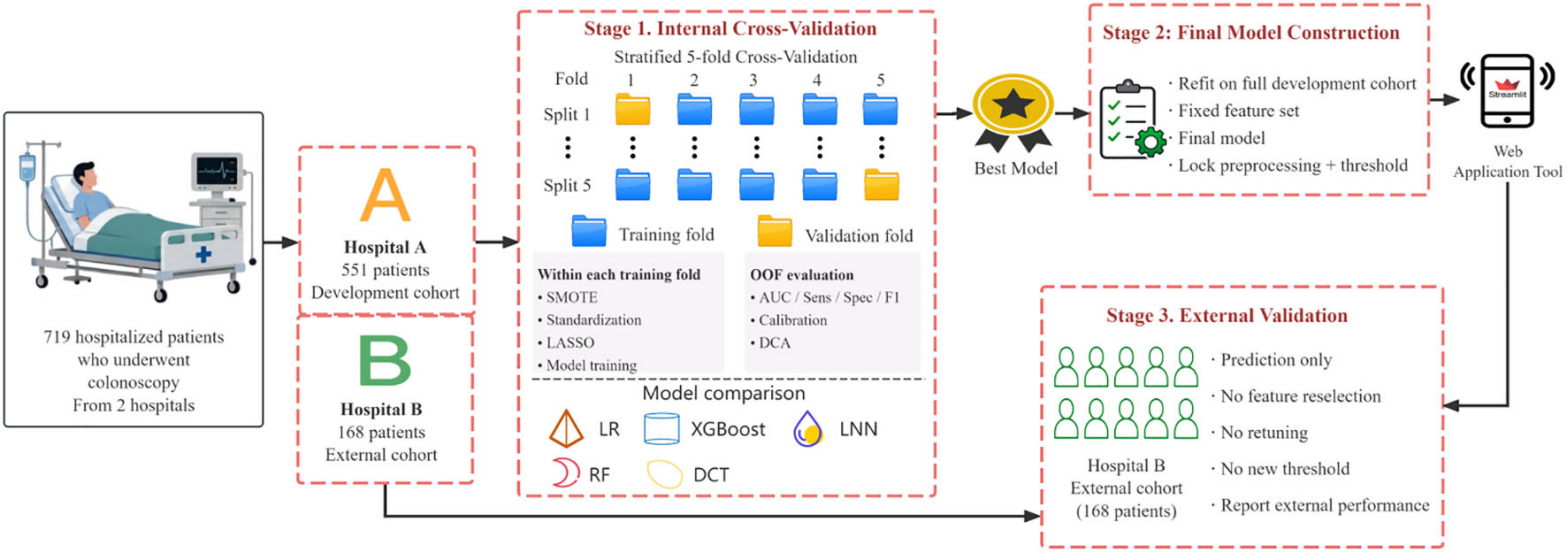
Workflow of web application development.

To facilitate clinical translation of the model, a cross-platform web application was developed based on the best-performing model using the Streamlit framework (v1.37.0) in a Python environment (**Figure 2**); the interface integrated native Streamlit UI components with an Ant Design–style design to enhance interactive friendliness. After clinicians enter a patient’s key predictor variables and click the “Predict” button, the predicted probability of peri-procedural hypoglycemia and the risk-stratification result for that patient are obtained in real time. To enhance interpretability, the tool integrated the SHAP library (v0.42.1) to automatically generate a real-time SHAP force plot, assisting clinicians in understanding the basis for the model’s individualized decisions. The tool supports both web and mobile access with good cross-platform compatibility, and its predictive efficacy was further validated independently on the external validation set (dataset B).

### 1.6 Statistical methods

Continuous variables were assessed for normality using the Shapiro–Wilk test: variables conforming to a normal distribution were expressed as mean ± standard deviation and compared between groups using the independent-samples t test; otherwise, they were expressed as median (interquartile range) and compared using the Mann–Whitney U test. Categorical variables were presented as frequency (%), and between-group differences were assessed using the χ² test or Fisher’s exact test. All tests were two-sided, and the significance level was set at P < 0.05. All data processing and statistical analyses were performed in Python 3.10.12.

## 2 Results

### 2.1 Baseline characteristics

According to the predefined inclusion and exclusion criteria, a total of 719 hospitalized patients undergoing colonoscopy were ultimately included, comprising 551 in the development set and 168 in the external validation set. During the study period, 109 peri-procedural hypoglycemic events occurred, with an overall incidence of 15.2%—82 (14.9%) in the development set and 27 (16.1%) in the external validation set; the difference in hypoglycemia incidence between the two datasets was not statistically significant (χ² = 0.142, P > 0.05). A total of 28 candidate predictor variables were included, and the baseline distribution of each variable was balanced between the two datasets (P > 0.05), as detailed in **Table 1**.

**Table 1:** Baseline characteristics of patients in the development and external validation cohorts.

Characteristics	Development cohort (n=551)	External validation cohort (n=168)	Statistic	P-value
Sex, n (%)			χ²=0.05	0.817
Female	257 (46.64)	76 (45.24)		
Male	294 (53.36)	92 (54.76)		
DM, n (%)			χ²=0.52	0.471
No	392 (71.14)	114 (67.86)		
Yes	159 (28.86)	54 (32.14)		
Insulin use, n (%)			χ²=0.00	0.994
No	468 (84.94)	142 (84.52)		
Yes	83 (15.06)	26 (15.48)		
Nutritional risk, n (%)			χ²=0.67	0.413
No	359 (65.15)	103 (61.31)		
Yes	192 (34.85)	65 (38.69)		
Fasting duration, n (%)			χ²=0.17	0.683
≤12 h	389 (70.60)	122 (72.62)		
>12 h	162 (29.40)	46 (27.38)		
Diet type, n (%)			χ²=0.10	0.951
Low-residue/low-fiber	282 (51.18)	84 (50.00)		
Liquid	102 (18.51)	31 (18.45)		
Other	167 (30.31)	53 (31.55)		
BP volume, n (%)			χ²=0.07	0.788
≤3 L	498 (90.38)	150 (89.29)		
>3 L	53 (9.62)	18 (10.71)		
BP type, n (%)			χ²=1.62	0.444
PEG	242 (43.92)	78 (46.43)		
Sodium sulfate	209 (37.93)	55 (32.74)		
Mannitol	100 (18.15)	35 (20.83)		
CHD, n (%)			χ²=1.66	0.197
No	447 (81.13)	128 (76.19)		
Yes	104 (18.87)	40 (23.81)		
Smoking history, n (%)			χ²=0.05	0.825
No	423 (76.77)	131 (77.98)		
Yes	128 (23.23)	37 (22.02)		
Drinking history, n (%)			χ²=1.16	0.281
No	350 (63.52)	115 (68.45)		
Yes	201 (36.48)	53 (31.55)		
Previous colonoscopy, n (%)			χ²=0.00	0.947
No	413 (74.95)	127 (75.60)		
Yes	138 (25.05)	41 (24.40)		
Age, years, median [IQR]	48.00 [36.00, 63.00]	47.00 [36.75, 63.00]	Z=−0.01	0.994
BMI, kg/m², median [IQR]	22.90 [20.50, 26.00]	22.30 [20.10, 25.12]	Z=1.45	0.148
Resting SBP, mmHg, median [IQR]	130.00 [120.00, 140.00]	130.00 [120.00, 140.00]	Z=0.08	0.933
Albumin, g/L, median [IQR]	40.30 [34.80, 43.30]	40.90 [35.71, 43.52]	Z=−0.50	0.620
Fasting C-peptide, ng/mL, median [IQR]	1.83 [0.99, 2.54]	1.59 [0.78, 2.48]	Z=1.47	0.142
ALP, U/L, median [IQR]	108.20 [77.75, 158.00]	105.40 [72.35, 142.25]	Z=1.27	0.203
AST, U/L, median [IQR]	23.85 [15.95, 52.00]	17.83 [14.40, 40.97]	Z=1.43	0.152
Total bilirubin, μmol/L, median [IQR]	18.94 [7.68, 43.85]	15.52 [6.97, 41.73]	Z=1.30	0.194
Direct bilirubin, μmol/L, median [IQR]	5.80 [3.50, 12.50]	5.41 [3.30, 11.31]	Z=0.60	0.547
Total cholesterol, mmol/L, median [IQR]	4.81 [3.52, 6.96]	4.73 [3.40, 7.24]	Z=0.37	0.712
Triglycerides, mmol/L, median [IQR]	1.22 [0.95, 1.65]	1.21 [0.93, 1.65]	Z=0.34	0.733
UA, μmol/L, median [IQR]	354.40 [295.70, 439.00]	372.40 [298.75, 467.00]	Z=−1.40	0.160
Platelets, ×10^9^/L, median [IQR]	242.00 [179.00, 310.00]	243.00 [161.50, 306.25]	Z=0.52	0.601
Na, mmol/L, median [IQR]	139.26 [136.37, 141.37]	138.81 [136.34, 141.06]	Z=0.78	0.434
Creatinine, μmol/L, median [IQR]	63.88 [54.56, 72.70]	64.95 [56.21, 74.79]	Z=−0.88	0.380
BUN, mmol/L, median [IQR]	6.47 [4.58, 8.76]	6.51 [4.59, 8.55]	Z=−0.36	0.722

DM, diabetes mellitus; CHD, coronary heart disease; BP, bowel preparation; PEG, polyethylene glycol; SBP, systolic blood pressure; BMI, body mass index; UA, uric acid; ALP, alkaline phosphatase; AST, aspartate aminotransferase; BUN, blood urea nitrogen; IQR, interquartile range.

### 2.2 Feature selection

**Figure 3:**
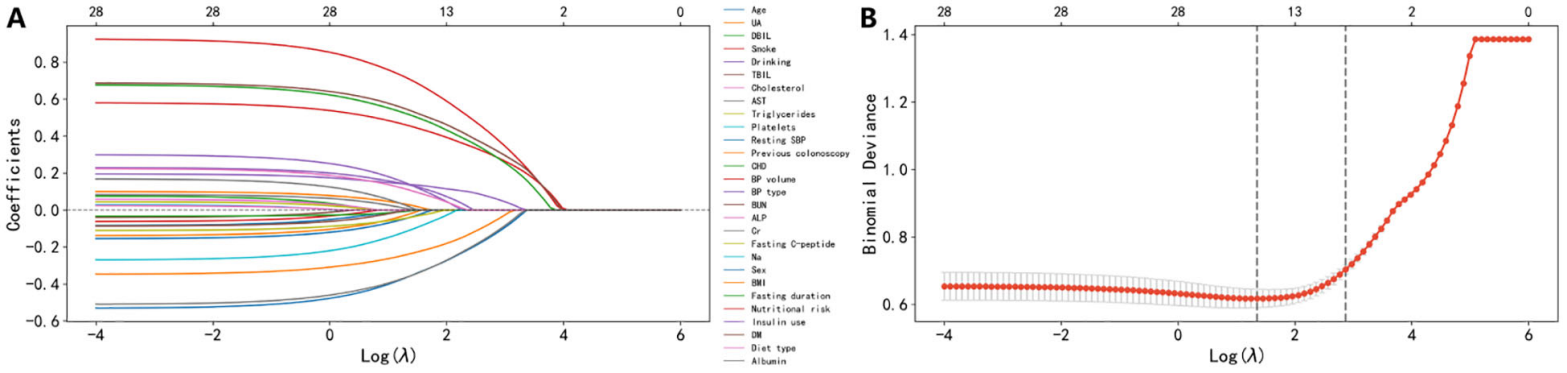
LASSO regression feature selection results. **(A)** LASSO coefficient shrinkage path plot; the x-axis is log(λ) and the y-axis is the regression coefficient of each candidate variable. **(B)** Five-fold cross-validation error curve; the x-axis is log(λ) and the y-axis is the binomial deviance. The left dashed line corresponds to λ_min and the right dashed line corresponds to λ_1se.

The LASSO regression algorithm was used to perform feature selection on the 28 candidate predictor variables in the development set (**Figure 3**). **Figure 3A** shows the shrinkage paths of the LASSO regression coefficients of each candidate feature as a function of log(λ); as λ increased, the coefficients were progressively shrunk toward zero and redundant variables were successively eliminated. **Figure 3B** shows the relationship between binomial deviance and log(λ). This study adopted the clinically common λ_1se criterion—selecting the most parsimonious model within one standard error—to minimize feature redundancy while maintaining predictive performance. At λ_1se, log(λ) = −1.532, corresponding to a mean deviance of 0.7781 (± 0.0100), and seven features with non-zero coefficients were ultimately selected from the candidate variables: bowel preparation solution volume (BP volume), sex, fasting duration, nutritional risk, insulin use, history of diabetes mellitus (DM), and albumin.

### 2.3 Performance comparison before and after SMOTE

**Figure 4:**
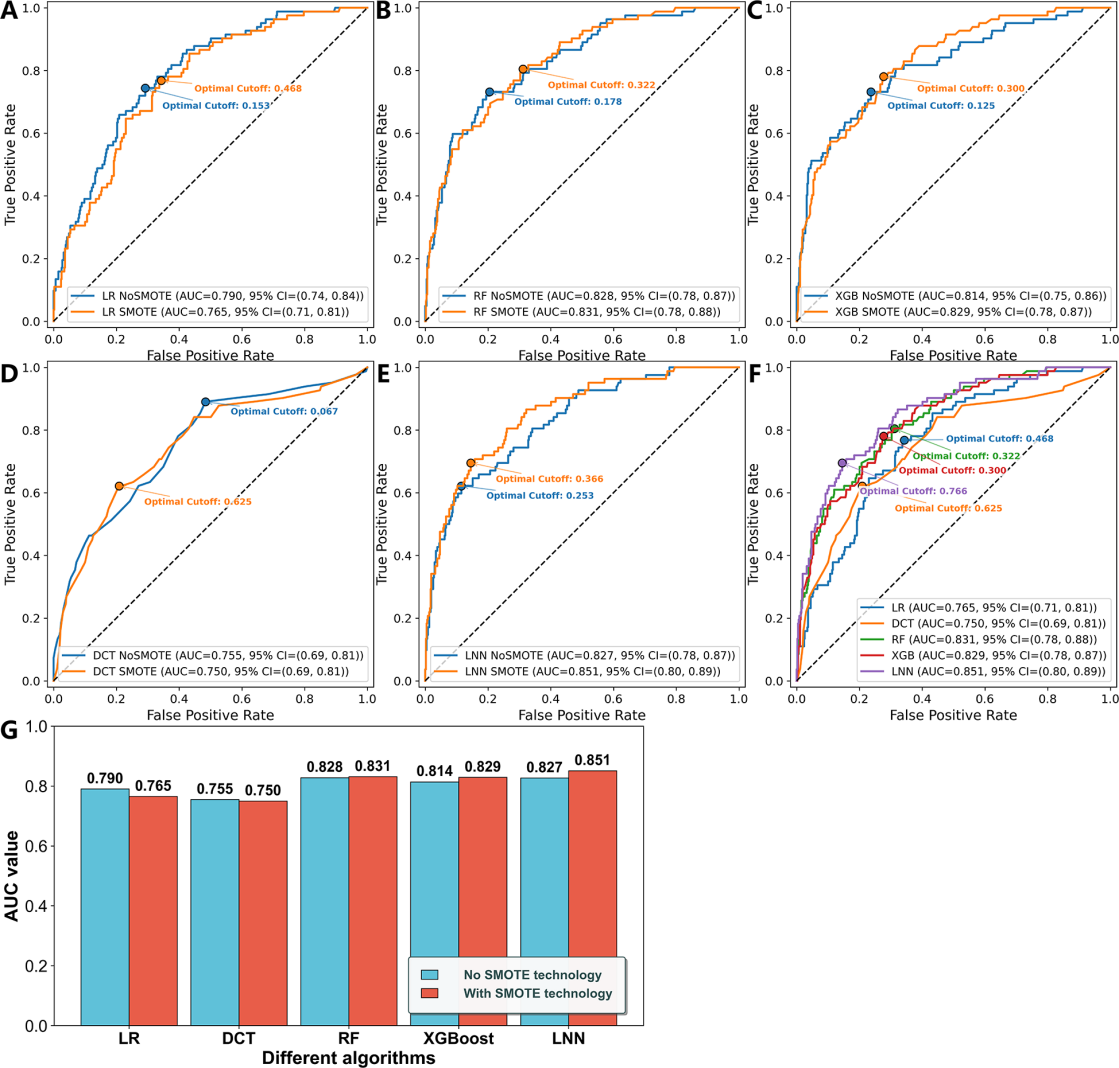
Comparison of the AUC values of the five models before and after SMOTE oversampling. **(A)** LR model; **(B)** RF model; **(C)** XGBoost model; **(D)** DCT model; **(E)** LNN model; **(F)** comparison of the AUCs of the five models after SMOTE; **(G)** comparison of the AUC values before and after SMOTE.

To evaluate the effect of class-imbalance handling on model performance, the change in AUC based on OOF predictions before and after applying SMOTE was compared across the five models (**Figure 4**). The effect of SMOTE on AUC varied across models: discrimination improved for LNN (0.827→0.851, ΔAUC = +0.024), XGBoost (0.814→0.829, ΔAUC = +0.015), and RF (0.828→0.831, ΔAUC = +0.003), whereas LR (0.790→0.765, ΔAUC = −0.025) and DCT (0.755→0.750, ΔAUC = −0.005) declined to varying degrees, with LNN obtaining the largest SMOTE gain.

The overall performance of the five models after SMOTE is shown in **Table 2**. The AUC of the LNN model was 0.851 (95% CI: 0.804–0.893), higher than that of RF (0.831), XGBoost (0.829), LR (0.765), and DCT (0.750); Pairwise comparison of the ROC curves using DeLong’s test indicated that the LNN significantly outperformed LR (P < 0.001) and DCT (P < 0.001), whereas its AUC did not differ significantly from those of RF (P = 0.20) or XGBoost (P = 0.16). Its specificity (85.50%), accuracy (84.21%), PPV (48.09%), and F1 score (59.15%) ranked first among the five models, and its sensitivity (76.83%) was second only to RF (80.49%), demonstrating the best sensitivity–specificity balance. The LR model had a specificity of only 65.67% and a PPV of 27.80%, with a high false-positive rate and the weakest overall performance. The optimal classification cutoff threshold of the LNN model was determined to be 0.366 by maximizing the Youden index on the development set alone; this threshold was then applied unchanged to the external validation set. Clinically, this cut-off favors a high specificity and a high negative predictive value, prioritizing the reliable exclusion of low-risk patients at the cost of a moderate positive predictive value, a trade-off that is acceptable given that intensified glucose monitoring and nutritional intervention are low-risk, low-cost measures.

**Table 2:** Internal-validation performance of the five models based on out-of-fold prediction after SMOTE within the training folds.

Model	Sensitivity (%)	Specificity (%)	Accuracy (%)	PPV (%)	NPV (%)	F1 (%)	AUC [95% CI]
LR	75.61[65.31–83.62]	65.67[61.26–69.83]	67.15[63.12–70.94]	27.80[22.34–34.02]	93.90[90.77–96.02]	40.66[33.56–47.42]	0.765 [0.711–0.815]
DCT	69.51[58.86–78.42]	83.58[79.96–86.66]	81.49[78.03–84.51]	42.54[34.49–51.00]	94.00[91.30–95.91]	52.78[44.55–60.56]	0.750 [0.686–0.809]
RF	80.49[70.63–87.62]	78.25[74.30–81.75]	78.58[74.97–81.81]	39.29[32.22–46.83]	95.82[93.32–97.41]	52.80[45.04–60.08]	0.831 [0.779–0.875]
XGBoost	74.39[64.00–82.60]	76.33[72.28–79.96]	76.04[72.31–79.42]	35.47[28.70–42.86]	94.46[91.68–96.35]	48.03[40.30–55.36]	0.829 [0.779–0.872]
LNN	76.83[66.62–84.63]	85.50[82.03–88.40]	84.21[80.93–87.02]	48.09[39.71–56.58]	95.48[93.04–97.09]	59.15[51.18–66.67]	0.851 [0.804–0.893]

**Figure 5:**
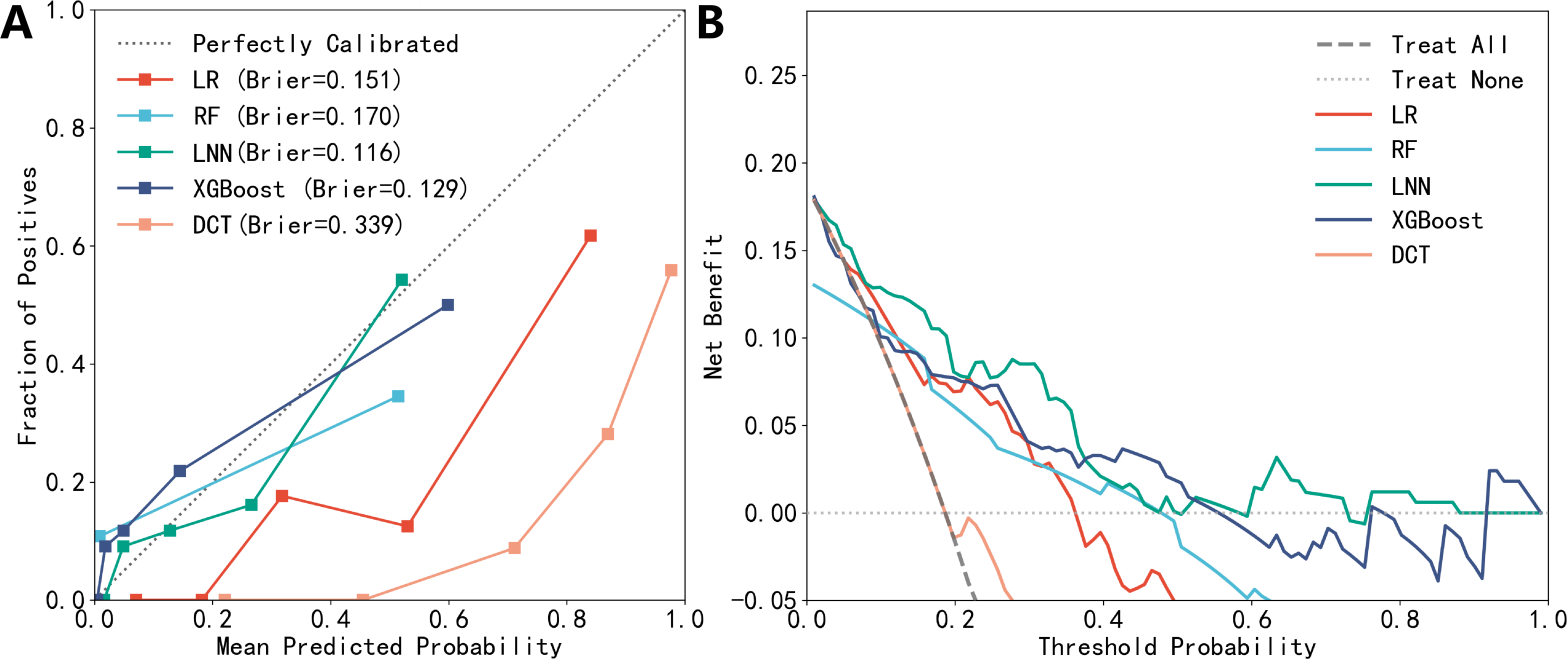
Internal-validation calibration curves and decision curve analysis of the five models based on out-of-fold predictions. **(A)** Calibration curves; **(B)** decision curve analysis (DCA).

To further evaluate clinical utility, calibration curves and decision curves were plotted (**Figure 5**). The LNN model had the lowest Brier score (0.116), better than XGBoost (0.129), LR (0.151), RF (0.170), and DCT (0.339), indicating the best agreement between predicted and observed probabilities; the DCT model showed the worst overall calibration, and the LR model markedly overestimated probabilities in the high-probability range (> 0.6). The DCA results showed that the LNN model consistently maintained the highest or near-highest net benefit across the main threshold-probability range of approximately 0.05–0.55; the net benefit of the DCT model fell rapidly below zero once the threshold probability exceeded approximately 0.20, and that of the RF model gradually fell below zero after approximately 0.45–0.50, indicating limited clinical value in higher-threshold decision scenarios. Considering discrimination, calibration, and clinical net benefit together, the LNN model performed best and was therefore selected as the final deployment model.

### 2.4 Web application development and external validation

**Figure 6:**
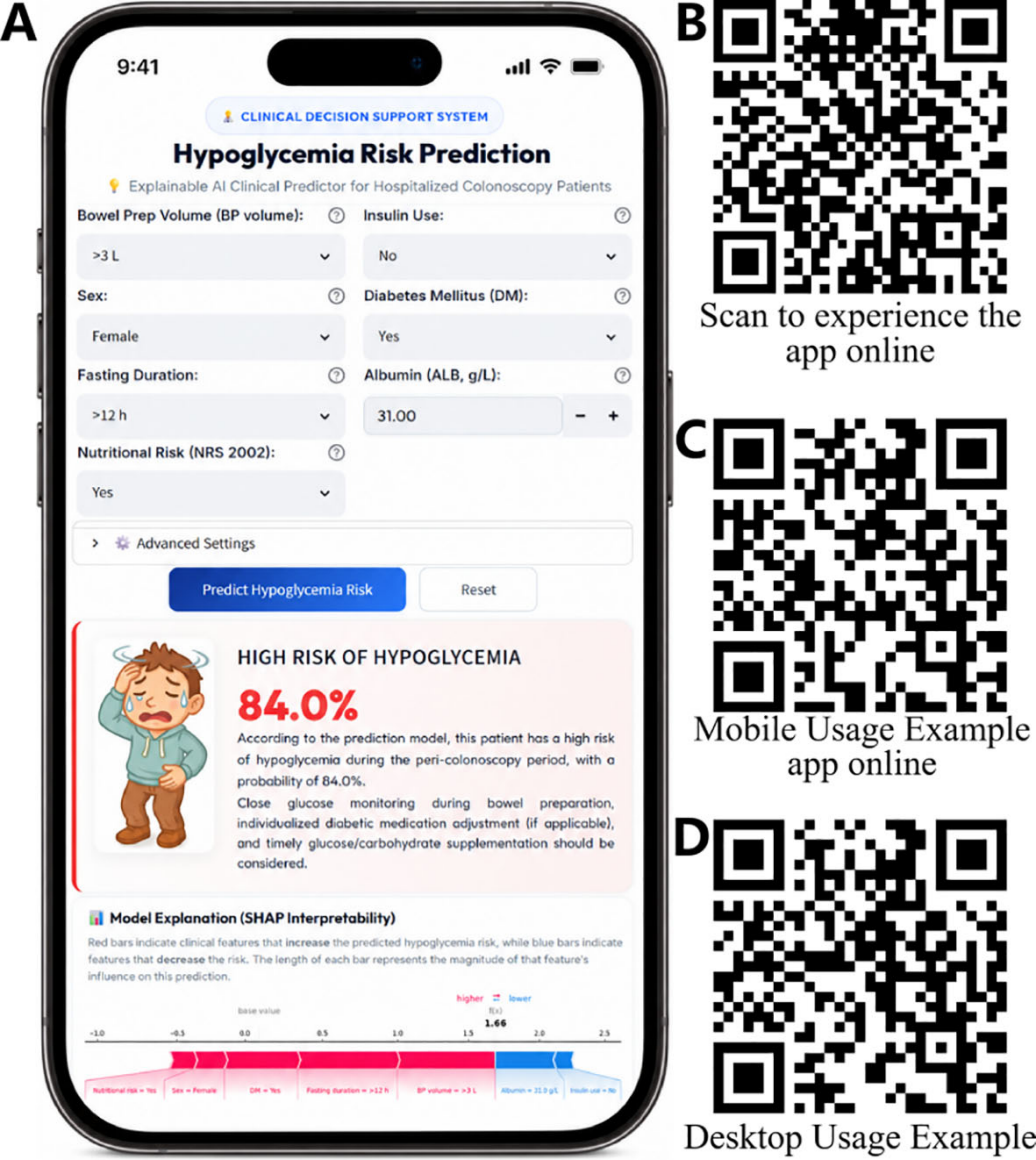
Web application for predicting peri-colonoscopy hypoglycemia risk developed based on LNN. **(A)** App interface; **(B)** scan the QR code to try the app online; **(C)** example of mobile use; **(D)** example of desktop use.

This study translated the LNN model with the best overall performance into an online prediction tool (**Figure 6A**) for assessing the risk of peri-procedural hypoglycemia in hospitalized patients undergoing colonoscopy. After healthcare providers enter the patient’s seven clinical parameters—bowel preparation solution volume (BP volume), sex, fasting duration, nutritional risk, insulin use, history of diabetes mellitus (DM), and albumin—and click the “Predict” button, the patient’s peri-procedural hypoglycemia risk level, specific probability value, and an individualized feature-contribution force plot based on SHAP values are obtained in real time. In the force plot, red indicates features that increase hypoglycemia risk and blue indicates features that decrease risk, enabling clinicians to intuitively understand the basis of the model’s decisions. The tool supports access on all platforms; users can quickly run it on mobile (**Figure 6C**) and desktop (**Figure 6D**) devices by scanning a QR code (**Figure 6B**).

**Figure 7:**
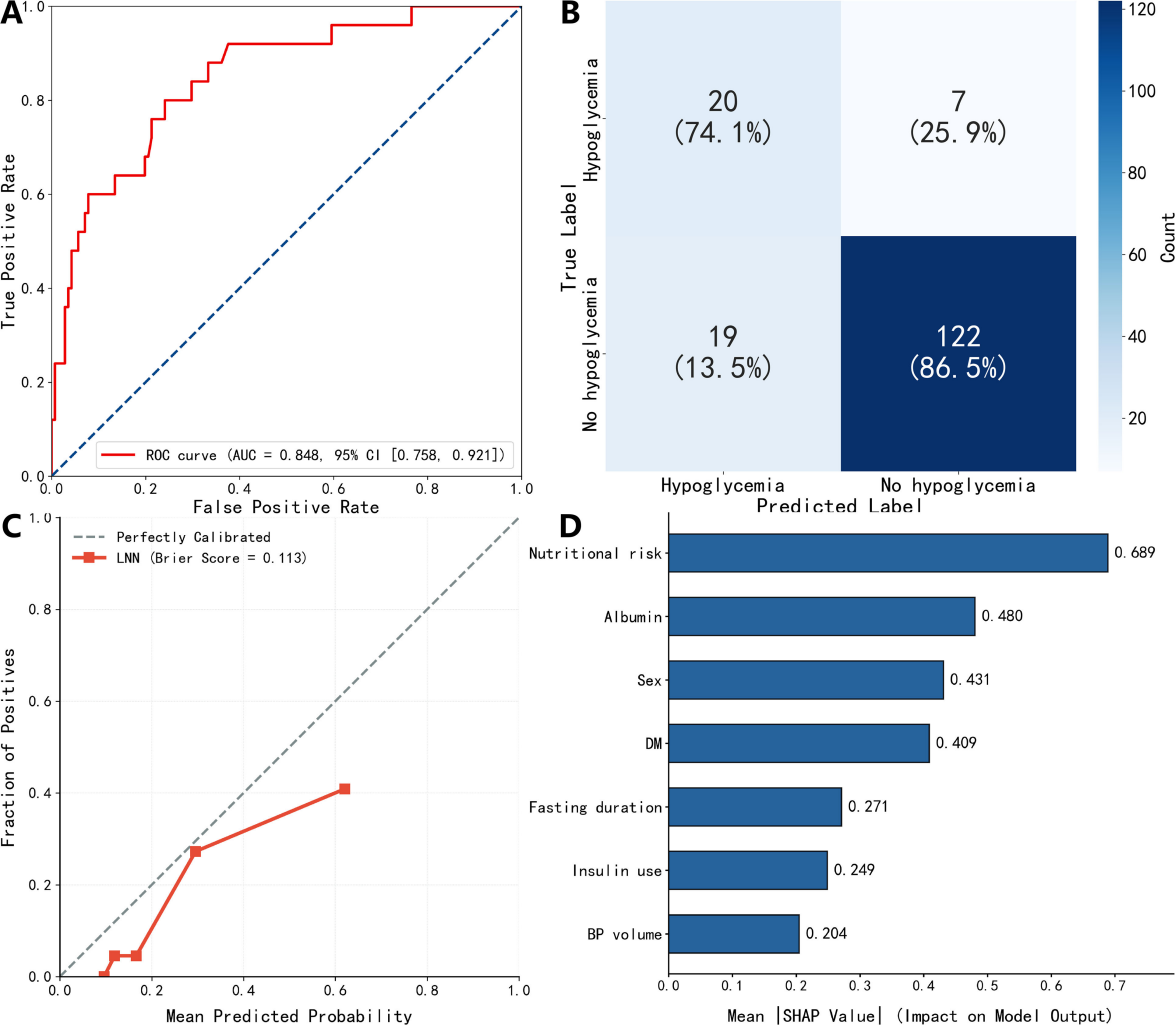
Performance evaluation of the LNN-based point-of-care tool on the external validation set. **(A)** ROC curve; **(B)** confusion matrix; **(C)** calibration curve of the LNN model in the external validation cohort (Brier score = 0.113). **(D)** variable importance analysis.

In the external validation set of 168 patients, the AUC of the LNN model was 0.848 (95% CI: 0.758–0.921), as shown in **Figure 7A**. Specifically, 20 of 27 hypoglycemia-positive patients were correctly identified, and 122 of 141 non-hypoglycemia patients were accurately classified, with the corresponding confusion matrix shown in **Figure 7B**. The model’s sensitivity was 74.07% (95% CI: 55.32–86.83), specificity 86.52% (95% CI: 79.91–91.20), accuracy 84.52% (95% CI: 78.29–89.21), positive predictive value 51.28% (95% CI: 36.20–66.14), negative predictive value 94.57% (95% CI: 89.22–97.35), and F1 score 60.61% (95% CI: 44.77–73.98). The model also remained well calibrated in the external cohort, with a Brier score of 0.113 (close to the internal-validation value of 0.116) and a calibration curve broadly following the diagonal of perfect agreement, with only modest overestimation in the highest predicted-probability range (**Figure 7C**). Feature importance analysis (**Figure 7D**) showed that nutritional risk was the most important predictor (mean |SHAP value| = 0.689), followed by albumin (0.480), sex (0.431), history of diabetes mellitus (DM, 0.409), fasting duration (0.271), insulin use (0.249), and bowel preparation solution volume (BP volume, 0.204). In summary, the LNN model demonstrated good generalization on the external validation set, with an AUC of 0.848, and its high specificity (86.52%) and negative predictive value (94.57%) indicate good clinical value in ruling out patients at low risk of hypoglycemia.

**Figure 8:**
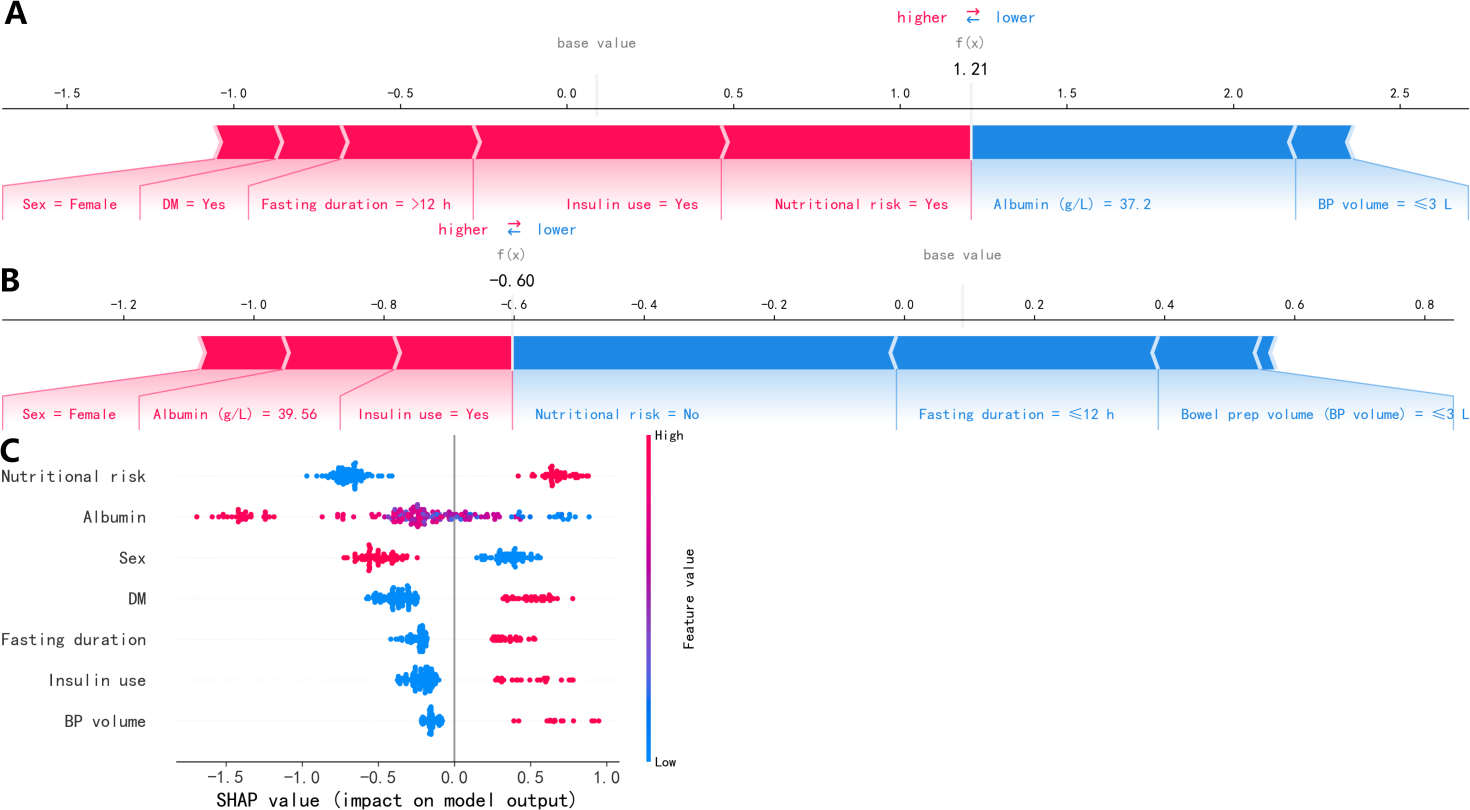
SHAP interpretability analysis of the LNN model. **(A)** SHAP force plot of a patient who actually experienced hypoglycemia; the model predicted a hypoglycemia probability of 82.02%, classified as high risk. **(B)** SHAP force plot of a patient who did not experience hypoglycemia; the model predicted a 65.00% probability of no hypoglycemia, classified as low risk. **(C)** SHAP summary plot of the LNN model on the external validation set, showing the overall contribution and directional distribution of each predictor variable to the model output.

To intuitively illustrate the individualized decision process of the LNN model, two cases were randomly selected from the external validation set for SHAP force-plot analysis (**Figure 8**). **Figure 8A** shows a patient who actually experienced hypoglycemia. For this patient, positive nutritional risk (nutritional risk = Yes), insulin use (insulin use = Yes), fasting duration > 12 h (fasting duration = >12 h), positive history of diabetes (DM = Yes), and female sex (sex = Female) were the main positive drivers—that is, factors increasing the tendency toward hypoglycemia—whereas albumin (albumin = 37.2 g/L) and bowel preparation solution volume ≤ 3 L (BP volume = ≤3 L) had a negative effect on the prediction. Taking these factors together, the model output a predicted hypoglycemia probability of 82.02% for this patient, classified as high risk, consistent with the actual outcome. Applying the same approach to the case in **Figure 8B**, the model predicted a 65.00% probability of no hypoglycemia, classified as low risk, consistent with the actual outcome. **Figure 8C** shows the SHAP summary plot of the LNN model on the external validation set, revealing at the global level the direction and magnitude of each feature’s effect on hypoglycemia prediction.

## 3 Discussion

Based on a two-center retrospective cohort, this study constructed and compared five models—LR, DCT, RF, XGBoost, and LNN—and developed a Streamlit web application integrating real-time SHAP interpretation. The results showed that LNN maintained good discrimination in both internal validation (AUC = 0.851) and external validation (AUC = 0.848), together with low Brier scores (0.116 internally and 0.113 externally) and high clinical net benefit; the negative predictive value in external validation was 94.57%, indicating that the model is particularly suitable for assisting in the exclusion of low-risk patients.

LASSO ultimately selected seven variables—bowel preparation solution volume, sex, fasting duration, nutritional risk, insulin use, history of diabetes mellitus, and albumin—broadly covering the three risk pathways of “reduced intake–insufficient reserve–drug effect.” Dietary restriction and clear-liquid intake associated with bowel preparation can affect glycemic stability, and patients with diabetes must additionally contend with issues such as adjustment of glucose-lowering agents and altered gastrointestinal motility (2, 3, 18); prolonged fasting and insulin use further increase the risk of hypoglycemia (6, 7). Nutritional risk and albumin reflect baseline nutritional reserve; previous studies in hospitalized patients have shown that a positive NRS 2002 result is associated with an increased risk of hypoglycemia in women, whereas higher albumin levels are associated with a reduced risk of hypoglycemia (19), consistent with the importance ranking of nutritional risk, albumin, and sex in this study.

SHAP analysis provided supplementary evidence for the directional interpretation of these variables. In the external validation set, nutritional risk, albumin, sex, and history of diabetes had the highest mean absolute SHAP values, indicating that the model does not rely on a single diabetes-related variable alone but comprehensively reflects the patient’s nutritional status, peri-procedural preparation behavior, and metabolic baseline. The individual SHAP force plot can also show the factors increasing or decreasing risk for each patient, helping the nursing team to tailor fasting management, energy supplementation, and the frequency of blood glucose monitoring (14, 20).

Compared with previous models for predicting peri-colonoscopy hypoglycemia, the main contributions of this study are the inclusion of both diabetic and non-diabetic hospitalized patients, the multi-algorithm comparison, and the completion of independent external validation. Yang et al. (6) and Lu et al. (7) established logistic regression models in patients with diabetes, with AUCs of approximately 0.777–0.82; the external-validation AUC of 0.848 in this study may be related to more comprehensive coverage of candidate variables, the strong complementarity of variables after LASSO selection, and the capacity of LNN to fit nonlinear relationships and variable interactions (12, 13, 21). However, because these studies differ substantially from ours in patient populations, predictor sets, study designs, and validation procedures, such cross-study comparisons should be interpreted with caution and should not be taken as evidence of the direct superiority of one model over another on the basis of AUC alone; the added value of the present work lies primarily in its broader patient coverage and its more complete evaluation of discrimination, calibration, and clinical net benefit, including independent external validation. Within the present internal comparison, DeLong’s test indicated that the LNN’s AUC was significantly higher than those of LR and DCT (both P < 0.001) but did not differ significantly from those of RF or XGBoost (P = 0.20 and P = 0.16, respectively); the comprehensive advantage of the LNN therefore rested on its concomitantly lowest Brier score (0.116) and highest clinical net benefit on DCA, rather than on discrimination alone. It should be noted that, given that this study used static tabular data obtained at admission, the main purpose of introducing LNN was not to exploit its temporal-modeling capability but rather to serve as an exploratory methodological validation, aiming to test whether the continuous-time architecture fits static nonlinear relationships and variable interactions better than traditional tree-based models; at the same time, this architectural choice also reserves room for future incorporation of time-series data—such as continuous dynamic blood glucose and real-time vital signs—to enable dynamic risk assessment.

Class-imbalance handling is an aspect of this study that requires cautious interpretation. The hypoglycemia incidence in the development set was 14.9%, and SMOTE was performed only within the training folds while the validation folds and external validation set retained their original distribution, so as to reduce the risk of data leakage and performance overestimation. The results showed that the effect of SMOTE was inconsistent across models: the AUCs of LNN, XGBoost, and RF improved slightly, whereas those of LR and DCT decreased. Previous simulation studies suggest that oversampling methods may improve some classification metrics but can cause overestimation of minority-class risk probabilities and worsen calibration (22). In other words, synthetic oversampling may inflate apparent discrimination while degrading probability calibration and, potentially, generalizability to populations with the original event rate; this is why SMOTE was confined to the training folds and the validation folds and external set retained their native prevalence. Therefore, this study did not select the model based on AUC alone, but simultaneously compared calibration curves, Brier scores, and DCA; this is also more consistent with the principle that clinical prediction models should comprehensively report discrimination, calibration, and clinical net benefit (23, 24). Reassuringly, the finally selected LNN achieved the lowest Brier score, suggesting that, under the present pipeline, the impact of SMOTE on calibration was limited.

From the perspective of clinical application, this tool requires only seven routine indicators as input to output a risk probability, risk stratification, and an individualized SHAP interpretation, making it more convenient for bedside use than traditional nomograms or offline models. The high negative predictive value suggests that it can be used to identify low-risk populations and reduce unnecessary high-intensity monitoring; for high-risk patients, it can prompt earlier optimization of the bowel preparation regimen, shortening of unnecessary fasting time, and intensified blood glucose monitoring and nutritional intervention. However, this tool has not yet undergone a prospective impact study, and whether it can truly reduce the incidence of hypoglycemia, decrease examination delays, or improve nursing workload remains to be further verified.

This study has the following limitations. First, as a two-center retrospective study confined to a single province and restricted to inpatients who completed standard bowel preparation and colonoscopy with relatively complete records, it may entail regional and selection bias and under-represent more complex or incompletely documented cases; larger multicenter prospective cohorts are needed to confirm generalizability. Second, the external validation cohort included only 27 hypoglycemic events, which widens the confidence intervals of event-related metrics, limits the precision and robustness of the performance estimates, and precluded robust subgroup analyses (e.g., diabetic vs. non-diabetic patients or insulin users vs. non-users); the model has not yet been assessed for recalibration in independent populations or validated across geographically distinct environments. These external-validation findings should therefore be regarded as preliminary and exploratory, and warrant confirmation in larger, multicenter prospective studies before any definitive claim about the model’s generalizability and robustness can be made. Third, all variables were static baseline indicators obtained at admission, which did not fully exploit the advantages of LNN in modeling time-series and irregularly sampled data; future work could incorporate dynamic variables such as continuous glucose monitoring, glucose-lowering regimen adjustments, and real-time vital signs to enable more precise dynamic risk assessment.

The LNN-based model demonstrated good predictive performance for assessing peri-procedural hypoglycemia risk in hospitalized patients undergoing colonoscopy and maintained satisfactory discrimination and calibration during external validation. The accompanying web application with real-time SHAP-based interpretation provides an interpretable and individualized decision-support tool that may facilitate early risk stratification. However, the model should be applied with appropriate clinical caution, as it was developed and validated using a relatively limited retrospective dataset. Larger prospective, multicenter validation studies are necessary to confirm its robustness, generalizability, and clinical utility before widespread implementation in routine practice.

## Data Availability

The raw data supporting the conclusions of this article will be made available by the authors, without undue reservation.
