# Supplementary material for: Development of an interpretable machine learning model and web application for peri-colonoscopy hypoglycemia risk in hospitalized patients undergoing colonoscopy
Source: Front Endocrinol (Lausanne). 2026 Jul 20;17:1903407. doi: 10.3389/fendo.2026.1903407 (PMC13429476; doi:10.3389/fendo.2026.1903407)
Supplement: Supplementary file 1 [file Table1.docx]

**Supplementary Table S1. Proportion of missing values for each candidate predictor before imputation.**

| Predictor | Variable type | Development cohort (n = 551), missing n (%) | External validation cohort (n = 168), missing n (%) | Overall cohort (n = 719), missing n (%) |
| --- | --- | --- | --- | --- |
| Age | Continuous | 0 (0.00) | 0 (0.00) | 0 (0.00) |
| Sex | Categorical | 0 (0.00) | 0 (0.00) | 0 (0.00) |
| BMI | Continuous | 7 (1.27) | 1 (0.60) | 8 (1.11) |
| DM | Categorical | 0 (0.00) | 0 (0.00) | 0 (0.00) |
| CHD | Categorical | 2 (0.36) | 0 (0.00) | 2 (0.28) |
| Previous colonoscopy | Categorical | 3 (0.54) | 2 (1.19) | 5 (0.70) |
| Smoking history | Categorical | 5 (0.91) | 1 (0.60) | 6 (0.83) |
| Drinking history | Categorical | 4 (0.73) | 2 (1.19) | 6 (0.83) |
| Albumin | Continuous | 13 (2.36) | 3 (1.79) | 16 (2.23) |
| Fasting C-peptide | Continuous | 45 (8.17) | 10 (5.95) | 55 (7.65) |
| Total bilirubin | Continuous | 16 (2.90) | 7 (4.17) | 23 (3.20) |
| Direct bilirubin | Continuous | 18 (3.27) | 5 (2.98) | 23 (3.20) |
| AST | Continuous | 14 (2.54) | 6 (3.57) | 20 (2.78) |
| ALP | Continuous | 15 (2.72) | 6 (3.57) | 21 (2.92) |
| Total cholesterol | Continuous | 23 (4.17) | 6 (3.57) | 29 (4.03) |
| Triglycerides | Continuous | 20 (3.63) | 9 (5.36) | 29 (4.03) |
| BUN | Continuous | 15 (2.72) | 4 (2.38) | 19 (2.64) |
| Creatinine | Continuous | 12 (2.18) | 5 (2.98) | 17 (2.36) |
| Na | Continuous | 13 (2.36) | 3 (1.79) | 16 (2.23) |
| UA | Continuous | 20 (3.63) | 4 (2.38) | 24 (3.34) |
| Platelets | Continuous | 8 (1.45) | 4 (2.38) | 12 (1.67) |
| Resting SBP | Continuous | 6 (1.09) | 1 (0.60) | 7 (0.97) |
| BP volume | Categorical | 1 (0.18) | 2 (1.19) | 3 (0.42) |
| BP type | Categorical | 1 (0.18) | 0 (0.00) | 1 (0.14) |
| Fasting duration | Categorical | 8 (1.45) | 1 (0.60) | 9 (1.25) |
| Nutritional risk | Categorical | 14 (2.54) | 7 (4.17) | 21 (2.92) |
| Insulin use | Categorical | 3 (0.54) | 0 (0.00) | 3 (0.42) |
| Diet type | Categorical | 9 (1.63) | 2 (1.19) | 11 (1.53) |

*Abbreviations: DM, diabetes mellitus; CHD, coronary heart disease; BP, bowel preparation; SBP, systolic blood pressure; BMI, body mass index; Na, sodium; UA, uric acid; ALP, alkaline phosphatase; AST, aspartate aminotransferase; BUN, blood urea nitrogen.*
